# Parental Beliefs about Childhood and Adolescence from a Longitudinal Perspective

**DOI:** 10.3390/ijerph18041760

**Published:** 2021-02-11

**Authors:** Pilar Ridao, Isabel López-Verdugo, Carmen Reina-Flores

**Affiliations:** Faculty of Education Sciences, University of Seville, 41013 Sevilla, Spain; pilridao@us.es (P.R.); mcreina@us.es (C.R.-F.)

**Keywords:** parental beliefs, parenting styles, psychosocial adjustment, longitudinal perspective

## Abstract

Research into family context as a socializing agent points to the need to take parental beliefs into account due to the role they play in both parenting strategies and, ultimately, in the psychosocial adjustment of children and adolescents. The present study aims to explore possible relationships between parental beliefs about childhood and adolescence from a longitudinal and qualitative perspective. The beliefs held by parents of teenagers about adolescence are compared with those they hold about childhood at that same moment, and the evolution of these ideas is charted over the course of 16 years as their children grow. A total of 102 parents participated in the longitudinal study. They completed two types of semi-structured interviews: one of them throughout the entire study period and the other once their children became teenagers. The results reveal an association between the type of beliefs parents hold about childhood and their perception of adolescence, and they indicate that these ideas change over time as more adjusted and modern beliefs about child development correlate with a more positive perception of adolescence. These results are interpreted from the perspective of their influence on beliefs about parenting styles, reflecting what is reported in the recent literature regarding the most successful styles for fostering children’s and adolescents’ psychosocial adjustment.

## 1. Introduction

Of the many factors that influence family dynamics, parental ideas or beliefs about children’s development and upbringing constitute the basis of parenting and provide a scaffold for determining how and why children act in a certain way [1,2]. Parental ideas and beliefs, therefore, influence children’s development and adjustment [3]. Just like any other cognitive conception or construct, parental beliefs are influenced by the social and cultural environment in which they are generated. We live in an era influenced by globalization and migration, in which families are forced to adapt to social and cultural conditions that are constantly changing [3] and act as priorities driving this adaptation.

After the study of parental beliefs took culture into consideration, Baumrind’s traditional model of parenting styles [4,5,6] was superseded by domain-specific models, which hold that parents adapt their parenting practices in accordance with their interpretation of parenting and child-rearing scenarios. Thus, the influence of parenting on children’s development and adjustment is mediated by values and ideas inherent to a specific context at a specific moment [7,8]. The system of ideas tends towards continuity although it can adapt to new circumstances [9,10], which may be triggered by elements outside the family system such as economic recessions or social crises like the current COVID-19 pandemic; however, these circumstances may also be triggered by the evolution of the family system itself and changes linked to the developmental stages of its members, particularly children. The present study focuses specifically on this second type of change in parental ideas by analyzing whether the beliefs of a group of parents from the same social and cultural environment are influenced by the different developmental stages of their children. The analysis was carried out from a two-fold applied perspective. Firstly, it is based on the premise that not all ideas are equally adaptive or have the same consequences for development. Thus, parents with a better knowledge of child-rearing and development tend to feel more satisfied with their parenting, make more internal attributions of their parental achievements and employ more positive parenting practices, all of which have a positive impact on their children’s adjustment [10,11]. Secondly, parental beliefs are considered key components in family interventions, acting as natural resources or assets for promoting more optimal parenting styles in accordance with social and cultural contexts of origin [12,13].

### 1.1. Parenting Strategies

Classic studies on parenting styles fall within the framework of two research traditions. One, initiated by Baumrind, establishes different parenting categories or styles: indulgent, authoritative, authoritarian and neglectful [4,5,6,14]; the other, advocated by Darling and Steinberg [15,16], proposes an integrative model of parenting styles and strategies in which greater importance is attached to styles.

Although data regarding which parenting style is the more appropriate are inconclusive, general agreement does exist in relation to which parental socialization styles influence psychosocial adjustment during childhood and adolescence [7,17,18,19,20,21]. In general, research in this field has followed the parenting style framework [22,23] and traditionally found that the style that combines affection and supervision (authoritative parenting) is the one that fosters the highest level of psychosocial adjustment among children. However, an emerging set of studies carried out in different social and cultural environments has recently begun to question the benefits of the authoritative model in comparison with the indulgent one by uncovering important inconsistencies in relation to this conclusion and presenting powerful empirical evidence that indulgent parenting is the most beneficial for sociopersonal adjustment [24,25,26,27,28].

Studies carried out in the English-speaking context with ethnic minorities (the Afro-American, Asian and Arab communities) suggest that a parenting style characterized by severity and a lack of affection (authoritarian) results in good child development, since in these cultures authoritarian practices are associated with parental commitment and responsibility [29,30]. Similar results have been found in research conducted in high conflict and psychosocial risk areas in which authoritarian strategies act as a protective factor for children’s adjustment [31,32].

However, studies carried out in Latin countries (Peru, Brazil, Italy, Portugal, Spain and Mexico) have found that the indulgent style is the more beneficial for both adjustment and development in all stages [26,33,34,35]. Similar results have also been reported in countries with different sociocultural characteristics from those found in Latin nations such as Sweden, Slovenia, the Czech Republic, the United Kingdom, Germany, Turkey and Norway [36,37,38,39].

Parental socialization styles are constructed and reconstructed on the basis of parental ideas about how children develop and should be raised [7], with these conceptions in turn being rooted in the sociocultural framework of reference [33,40,41,42]. Parental beliefs constitute a general outline of what is considered important in child-rearing, whereas parenting practices are interpreted as the way in which these ideas are materialized and refer to how parents behave in order to fulfill their child-rearing or socializing function. The result is a framework of analysis in which parental cognitions are linked to parental socialization strategies, which in turn influence the child’s adjustment [7,41,43]. However, the relationship between parental beliefs and parenting styles is neither simple nor direct. An action or practice may be mediated by a combination of various ideas since other factors also influence parental behavior, including their personal characteristics, their children’s personal characteristics and developmental stage and the demands of the situation at any given moment [44,45].

### 1.2. Parental Beliefs

Parental beliefs are defined as a set of naive theories about developmental achievements: what is important for child-rearing and those aspects that influence child development [41]. However, these theories (which are mainly unconscious) do not come out of nowhere but are rather rooted in the parents’ cognitive systems, which in turn are based on their knowledge of development, previous experiences with parenthood in their close family or social environment, and culturally inherited habits and behavior [44].

Once established, this set of beliefs determines how mothers and fathers parent and play a key role in the family system and in the socialization of children and adolescents. It also offers parents an on-going, unconscious framework for both assessing their children’s behavior and providing them with stimulating, affectionate, restrictive and inductive interactions and development opportunities [13,40].

The literature contains several different theoretical models of how these cognitions are structured. For example, the phase or stage-based approach [46,47] analyzes ideas in accordance with their structure in each phase, proposing various levels of ideas with differing degrees of complexity.

For its part, the belief-systems approach [2] posits that parental beliefs are not independent from each other but rather form a complex systemic structure that serves as a guide for everyday parenting. Ideas are defined as cognitive frameworks, or schema, on the basis of which parents establish parenting goals and interpret their child-rearing experiences [7].

The team led by J. Goodnow [48] attaches more importance to cultural and social factors in the construction of parental ideas, viewing them as the result of cultural constructions about child development. Consequently, these ideas play a key role in the configuration of the child’s socializing context.

Based on empirical findings, some authors have proposed an approach centered on typologies [40,45,49]. According to this perspective, ideas are grouped into categories, or typologies, in accordance with how they view the following aspects of parenting and child-rearing: perceived influence on development, expectations regarding developmental milestones and the role of the mother and the father in child-rearing (among others). These typologies include parents with more current or modern conceptions of child development and education as well as those with more stereotyped or traditional ideas [45,50]. According to these authors, parents with modern ideas are characterized by an interactionist view of development, an awareness of their own influence on their children’s development, a preference for authoritative parenting practices and a high level of education. Parents with traditional ideas, in contrast, are defined by an innate conception of development, a low perception of their own influence on their children’s development, gender-stereotyped ideas, more authoritarian parenting practices, and a low level of education. While many studies over recent decades have explored these cognitions and their properties [1,2,41,51,52], most have focused on perceptions of the rearing and development of children, with fewer authors seeking to explore ideas about adolescence and still fewer analyzing the differences or similarities between the two developmental stages.

Studies exploring ideas about childhood usually focus on different issues than those analyzing how parents perceive adolescence in accordance with the specific characteristics of each developmental stage. For example, the determinants of development or developmental goals and expectations are classic contents in works focusing on childhood, as evident in the aforementioned studies on parental cognitions and their properties. During adolescence, however, other factors tend to be of more interest due to the specific characteristics of the development which takes place during this stage. Traditionally, most research into the parents’ perceptions of adolescence takes three key aspects into account: expectations regarding developmental milestones, focusing mainly on teenagers’ achievement of autonomy and maturity [53,54]; disciplinary strategies, mostly in reference to control, authority and supervision [55,56,57]; and perceptions of the stage itself, focused on determining to what extent ideas about adolescence are stereotyped [58,59,60,61].

Although these features are clearly different, all parental perceptions are in fact based on an interwoven system of ideas. In other words, while parents often hold a wide variety of specific ideas about how their children learn and develop throughout the different stages, there is also an underlying ideological substrate that bestows coherence and unity on these cognitions [2,7]. Thus, different ideas are, to a greater or lesser extent, mediated or interconnected by this underlying cognitive structure.

Following the approach based on typologies of ideas [45,50], the present study is a longitudinal exploration of the beliefs held by a group of parents throughout the course of their children’s development. The aim is to determine whether beliefs about child-rearing function as general guidelines regarding what is important in parenting despite the changes and adjustments inherent to the different stages through which the members of the family system pass. Specifically, the aim of the study is to explore parental beliefs about childhood and adolescence, acknowledging the peculiarities inherent to each stage, while at the same time analyzing the possible association between ideas about the two developmental moments, understood as ideas about different characteristics that are nevertheless based on and rooted in the same ideological substrate or cognitive structure. The study therefore has two specific aims: (1), To identify parental ideas about child-rearing and development throughout the course of their children’s development; (2), To analyze the relationship between parents’ ideas about childhood and their perceptions of adolescence. The results are analyzed in relation to the following initial hypotheses: (1), Parental beliefs tend to be stable over time; (2), The content of parents’ beliefs can change to adapt to the evolutionary moment of their children; and (3), Parents have more traditional ideas about adolescence than about childhood.

## 2. Materials and Methods

### 2.1. Participants

Families were recruited at the birth of their child from hospitals belonging to the public health service. A varied and socio-demographically representative sample of the Spanish population was obtained. Participation was voluntary and informed consent was obtained at the beginning of the study. Participants were duly informed of the characteristics of the study, the confidential nature of the information provided, and that it would be used exclusively for research purposes.

This study involved 102 parents who participated in all phases of a longitudinal research project covering a period of 16 years. Parents were interviewed at four different moments of their children’s development: Time 1 (birth), Time 2 (22 months), Time 3 (7 years), Time 4 (16 years).

**Figure 1:**
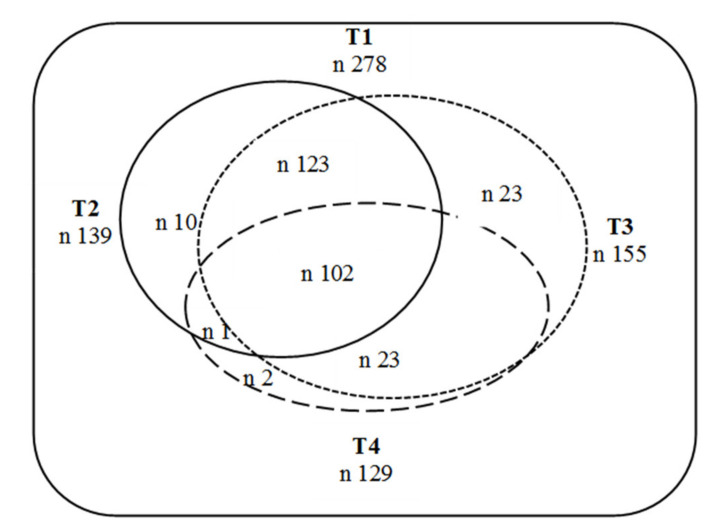
Sample composition at each time point of the longitudinal study.

The four time points were selected with the aim of collecting information about the principal developmental stages: infancy, preschool, school age and adolescence. The initial sample at Time 1 comprised 278 participants, whereas the sample at Time 4 comprised 129. Experimental mortality varied widely across the different phases of the study although it was the highest from Time 1 to Time 2. Only those parents who participated at all time points were included in the longitudinal comparisons. The sample composition at each moment is shown in Figure 1.

The sample comprised 55 mothers and 47 fathers. The distribution of the parents was compared in terms of their socio-demographic profile at the different time points with no statistically significant differences being found in the distribution of the variables: sex (χ^2^_(4)_ = 0.70, p = 0.95), habitat (χ^2^_(4)_ = 4.22, p = 0.38) and education level (χ^2^_(8)_ = 11.94, p = 0.15). As for education, 40.2% had a high education level, 31.4% a medium education level and 28.4% a low education level. Moreover, 35.3% lived in a rural habitat and 64.7% in an urban habitat. Upon accessing the study, 49.0% of participants were first-time parents, and by the last time point, 90.1% had more than one son or daughter. At Time 1, 43.1% were under 28 years of age, 46.1% were aged between 28 and 31 and 10.8% were over 31.

### 2.2. Instruments

Parental Ideas Questionnaire (Cuestionario de Ideas de Padres-CIP) [24]. The CIP assesses parental ideas about child-rearing and development during childhood by means of 39 questions. For the present study, we used only those questions that were found to be significant in previous studies [45,49,62] and were included at all time points of the longitudinal follow-up. The instrument is qualitative and is applied in the form of a semi-structured interview. In the first phase, it was administered to parents of newborns in the hospitals of the Andalusian public health system. In the subsequent phases of the study, interviews were held in the family home.

Based on all the answers given by participants during the interviews, a coding system was developed which included all response options grouped into different categories. Each response was assigned to a specific category. The same category system was maintained across all time points. Responses were coded nominally, with the coding systems developed being analyzed for inter and intra-rater reliability. The Kappa coefficients obtained were over 0.8.

Table 1 gives example questions from each dimension included in the two questionnaires (childhood and adolescence).

Parental Ideas about Adolescence Questionnaire (Cuestionario de Ideas sobre la Adolescencia-CIP-A) [63]. This instrument is a version of the previous one which assesses parents’ ideas about adolescence. It comprises 25 questions and gathers qualitative information by means of a semi-structured interview. It was only used during the final phase of the longitudinal follow-up, when the children were teenagers. The (inter and intra-rater) reliability assessment found a Kappa coefficient of over 0.8.

### 2.3. Data Analysis

A Multiple Correspondence Factor Analysis (MCFA) was carried out using the SPAD 3.5 statistical software package (Système Portable pour l’Analyse de Données, Paris, France) to determine the existence of some kind of structure or pattern in the ideas held by parents about childhood and adolescence. This analysis model is suitable for the multivariate processing of matrices with a large number of variables since it processes the information in an integrated manner. It is also recommended for data processing aimed at classifying and ordering a large number of variables and nominal variables [64,65]. This procedure reduces the dimensionality of the data matrix by extracting the factorial axes, which in this case are understood as groupings of parental ideas. These axes are formed by assigning a quantitative value to each type of response, with this value being an indicator of the importance of said type for interpreting the idea axis. Similarly, participants are also assigned a factorial score in accordance with the pattern of their response types. Once the factorial axes were extracted, participants were reclassified by means of a fragmentation analysis, which resulted in homogeneous groups of subjects.

In order to extract the idea typologies, a similar procedure was followed at each time point. The questions were treated as variables and the answer categories as modalities. The 39 questions of the CIP and the 25 questions of the CIP-A were selected as active variables for inclusion in the analysis. Sex, education level and habitat were used as illustrative variables to help enrich the information. Since the number of variables was high, it was necessary to apply the transformed autovalue. The factorial axes were then extracted and the relevant factors were selected for the description of the sample.

To determine the evolution of parental ideas over time, all change and continuity trajectories were identified throughout the course of the longitudinal follow-up, thereby enabling idea evolution patterns to be established. Evolution factors and profiles were then extracted from these change and continuity patterns using the MCFA method.

The idea patterns at each of the two developmental moments, as well as their evolution over time, were compared using the chi-squared association test offered by the SPSS (IBM Madrid, Spain) statistical program.

## 3. Results

### 3.1. Ideas about Childhood

In accordance with their ideas about childhood, parents can be divided into three main classes: Modern, Traditional–Moderate and Traditional (following the MCFA). These classes span the continuum between modernity and tradition, with two clearly identified as being at either end, and one being located in the middle, although slightly closer to the traditional extreme. Table 2 outlines the most representative types of answer given by parents in the different classes.

Parents in the Modern class (53.92%) held updated ideas about childhood, espousing beliefs that are similar to those expressed by experts in the field. Parents in this class generally have a high education level (v-test 3.45; *p* = 0.000; Type/Class 56.36) and are mainly mothers (v-test 1.93; *p* = 0.027; Type/Class 63.64).

Parents in the Traditional–Moderate (27.45%) and Traditional classes (18.63%) held more traditional ideas about childhood, with answers that contrasted with those espoused by parents in the Modern class. Although the majority of responses given by parents in the Traditional–Moderate class corresponded to the Traditional profile, some did not exactly coincide with this pattern (e.g., their assessment of their children’s initiative). The responses given by parents in the Traditional class are fully consistent with the traditional pattern, reflecting the idea that parents have little influence over their children’s development and strong gender stereotypes but no ideas that are closer to the more modern end of the spectrum. Education level was not found to be representative of these classes although a higher number of fathers was observed in the Traditional–Moderate class (v-test 2.50; *p* = 0.006; Type/Class 67.86). The principal contents for each class are shown in Table 2.

### 3.2. Ideas about Childhood throughout the Longitudinal Follow-Up

The patterns of parents’ evolution throughout the course of the longitudinal follow-up were analyzed and three types of pattern or trajectory were extracted: (1), those who remained constant in their modern ideas across the different time points (Stable Modern Pattern, 60%); (2), those who remained constant in their traditional ideas (Stable Traditional–Moderate Pattern, 23%); and (3), those whose ideas changed but within the traditional range (small variations in specific contents without moving too far from the traditional end of the spectrum) (Changing Traditional/Traditional–Moderate/Traditional–Moderate/Traditional Pattern, 18%). The configurations of each trajectory of evolving ideas about childhood are shown in Table 3.

Moreover, a chi-squared test was carried out to analyze the evolution of the ideas in accordance with sociodemographic variables. A significant association was observed between education level and evolution of ideas (χ^2^_(6)_ = 19.973; *p* = 0.003). Most parents in the Modern class whose ideas remained stable had a high education level with only a few having a low level. A smaller percentage of parents in the Stable Traditional–Moderate class had a high education level. In the Changing Traditional/Traditional–Moderate/Traditional–Moderate/Traditional class, the majority of parents had a low education level, with the percentage of those with a high level being small.

A significant association was also found between habitat and change–continuity patterns across the different time points (χ^2^_(3)_ = 8.806; *p* = 0.032). In the Stable Modern group, the percentage of parents living in an urban environment was higher, whereas in the Changing Traditional/Traditional–Moderate/Traditional–Moderate/Traditional class a higher percentage of parents lived in a rural context.

### 3.3. Ideas about Adolescence

Three types of ideas were identified by the MCFA: Modern (25.49%), Traditional–Moderate (53.92%) and Traditional (20.59%). Although the results in each class differed between childhood and adolescence, the same names were maintained because certain parallels were observed between them. Parents in the Modern class were found to hold more up-to-date ideas, and those in the Traditional–Moderate and Traditional classes more traditional ones with the traditional nature of beliefs being more marked in the latter.

The need for parents to be strongly involved with their adolescent children and the high level of maturity attributed to teenagers were the two most representative aspects of the Modern class. The perception of the stage as a difficult period characterized by tension and problems was the most striking aspect of the Traditional–Moderate class. Parents in this group also highlighted the importance of being involved with their teenage children, although to a lesser extent than their counterparts in the Modern group, and also ascribed to them a lower level of maturity. The Traditional class was mainly represented by fathers (v-test 2.89; *p* = 0.002; Type/Class 76.19), who held that the most important thing for teenagers is for them to have fun with their peers; parents in this class saw adolescents as being very immature. The principal responses for each class are shown in Table 4.

### 3.4. Comparison of Ideas at Different Developmental Moments

When comparing parents’ ideas about children with their ideas about adolescence, we saw that ideas about adolescence in the Modern group tended mainly to be the same for as for childhood and tended not to be in the Traditional group. For their part, parents who held Traditional ideas about adolescence also held Traditional ideas about childhood (X^2^_(4)_ = 16.514; *p* = 0.002). Table 5 shows the association between ideas about childhood and ideas about adolescence expressed in percentage terms.

The analysis of the results in terms of gender revealed that mothers with modern ideas about childhood (Modern) were more likely to have modern ideas also about adolescence (X^2^_(4)_ = 11.457, *p* = 0.022). For their part, fathers who had more traditional ideas about childhood (Traditional) mainly tended to have traditional ideas also about adolescence (X^2^_(4)_ = 9.503, *p* = 0.050).

When we compared idea classes with evolution patterns over time, we saw that parents in the Modern group for ideas about adolescence tended to form part of the Stable Modern Pattern, and those in the Traditional class for ideas about adolescence belonged to the Changing Traditional/Traditional–Moderate/Traditional–Moderate/Traditional pattern (X^2^_(4)_ = 10.176; *p* = 0.038).

## 4. Discussion

One of the characteristics and properties of parental beliefs is that they form a complex and dynamic system [2]. This is confirmed by the results presented here, which highlight the connection that exists between parents’ ideas about different developmental stages. 

The parents in our study have idea typologies that are consistent with those outlined in previous research. These ideas are organized along the dimension of modernity–traditionality [45,49,50,62]. Parents with more traditional beliefs about childhood express highly stereotypical ideas about development which are far removed from the needs of children in this stage. In contrast, those with more advanced and up-to-date views on child-rearing and developmental processes have a more optimistic view of development, which leads to parenting styles which foster better adjustment [10]. 

Regarding ideas about adolescence, the parents with more modern views highlighted maturity as a characteristic of adolescence. For their part, parents who hold more traditional views perceive adolescence as a stage characterized by a lack of commitment and oriented towards having fun. In other words, they highlight immaturity as a principal element of this stage, particularly at a socio-emotional level. In line with that reported by other authors, parental knowledge about child-rearing was found to affect parents’ views of adolescence, which in turn is linked to parenting styles and practices [3,66,67,68]. Whereas parents with traditional ideas subscribe to child-rearing models based on deficits, parents with more modern views base their beliefs on the model of positive adolescent development. These differences have an impact on adolescent development, fostering different levels of psychological adjustment in accordance with the different models espoused [69,70].

In terms of the position adopted by parents during the different stages of their children’s development, most parents adhere to more modern views in relation to childhood. However, during their children’s adolescence, parental beliefs about adolescence seem to respond to more traditional social patterns, which are dominated by issues linked to this age group and in which the influence of the social and cultural environment is more prominent [3]. 

A comparison of the ideas expressed by the participants regarding childhood and adolescence revealed that parents who consistently espoused more modern ideas have a positive perception of adolescence, while parents whose ideas change (within the traditional range of beliefs) tended to view this stage as characterized by immaturity and the search for enjoyment, which probably translates into parenting styles which are less stimulating and enriching for development. Like the scientific community, parents with more modern ideas seem to have moved beyond the Storm and Stress concept so often linked with adolescence [71,72,73]; however, this construct still appears to be endorsed by that part of the population that espouses more traditional ideas.

The findings reported here point to continuity of mental schemas during the different developmental stages studied [74], with parents who hold positive beliefs about childhood also having a more favorable image of adolescence. Within the general trend towards continuity, these changes imply an adaptation to the new circumstances of the environment [9]. Thus, parents’ views of adolescence are influenced by negative social constructions about the teenage years [75,76].

These results have clear practical implications for interventions with families, indicating that it is important to foster adapted ideas about childhood and to strive to ensure their continuity over time. It is also vital to debunk social myths and stereotypes about adolescence since they may influence the more traditional and stereotyped view held by many parents about this stage [71,77,78,79].

This training would help improve parenting practices by encouraging parents to foster children’s and adolescents’ competencies while at the same time helping them avoid risky behavior and the maladjustment to which practices based on traditional ideas may lead [80,81]. Indeed, recent research into family interventions advocates a model in which parents’ conceptions regarding child-rearing and development are viewed as a basic lever for optimizing parenting skills [80,82,83]. The promotion of positive development during adolescence, focused on teenagers’ competencies and potential, will enable parents to adopt a parenting style that results in better adjustment and improved well-being [84].

## 5. Conclusions

Parental ideas constitute a cognitive scaffold that underlies the way mothers and fathers parent their children. The results of the present study suggest that this scaffold is characterized as much by stability as by change. Parents tend to remain faithful to their ideas about child-rearing and development, staying within a certain type across the different developmental stages analyzed. However, at the same time, their ideas undergo changes in accordance with the developmental stage of their children, as well as the sociocultural changes experienced by the family system. In general, these changes indicate that parents have a more adapted knowledge of childhood, and more traditional and stereotyped ideas in relation to adolescence.

In sum, in accordance with current tendencies in the field of research into family context as a socializing agent, the idea that children’s psychosocial adjustment is mediated or moderated by parental beliefs is becoming increasingly important, and there is a growing awareness of the need to foster parenting skills that foster the adjustment of children and adolescents (rather than emphasizing maladjustment). It would be interesting to carry out family interventions during the childhood years to support an adapted view of this stage and promote a more positive, non-stereotyped perception of adolescence. Logically, such interventions should not be limited to parental ideas but should also strive to foster more effective parenting practices that are consistent with styles that promote the development and adjustment of children and adolescents [19,33]. Furthermore, it should not be forgotten that a family situation should always be interpreted within its specific sociocultural context [8].

Parental ideas start to be built prior to the birth of the child, making it advisable to address them before parenthood within those resources oriented towards its preparation [63].

Moreover, due to their stability and consistency, parental ideas are important elements for family intervention. Thus, in order to improve parental competencies and promote adjusted parenting styles, it is essential to approach parental ideas about development and education in childhood and adolescence [76,80].

This study has a number of limitations linked to both the research aims and the sample size, which are typical of longitudinal studies,. In relation to the former, while the properties of parental ideas were analyzed, their connection with parental socialization strategies and, ultimately, children’s psychosocial adjustment was not. In relation to the latter, it would have been better if the sample had been more representative of the ethnic and cultural minorities that live in our region to enable an assessment of the role played by sociocultural variables. In this sense, future studies may wish to assess which type of parental ideas and beliefs foster more adapted socialization strategies while taking sociocultural differences into account.

## Figures and Tables

**Table 1:** Contents discussed in the interviews about parents’ ideas regarding child-rearing and development during childhood and adolescence.

Dimension	Questions about Childhood (Parental Ideas Questionnaire, CIP)	Questions about Adolescence (Parental Ideas about Adolescence Questionnaire, CIP-A)
Ideas about developmental milestones	When do you think a child starts to talk well enough to be understood by strangers?	In what ways do you believe that a teenager the same age as your son/daughter is mature enough to, for example, have a girlfriend/boyfriend?
Nature or nurture	From a very young age, some children are more restless than others, some cry more and others are calmer. Why do you think children are so different from one another, right from when they are newborns?	Many people think that adolescence is a very difficult stage in which children start to act out, question their parents’ authority and become more irresponsible. Do you agree?
Parental role	Who do you think should be responsible for most childcare tasks both for babies and older children (e.g., bathing them, feeding them): the father, the mother or both?	I am going to describe a series of circumstances involving an adolescent in this age group, and I want you to tell me who you think should intervene: the father, the mother, both or neither: Example: Your teenage son or daughter arrives home at night two hours after the agreed-upon time.
Parent–child relationship	In what ways do you think there is a relationship between a pregnant woman and the fetus she is carrying?	I am going to read out a list of things that parents can do with their teenage children. I want you to tell me how important you think each thing is on a scale of 1 to 5: 1 = not at all important, and 5: very important/fundamental. Example: Get to know their friends.
Parents’ capacity to influence their children	Do you believe that parents can do something to foster their children’s intelligence?	Do you think that, at this age (the age of their son or daughter), parents should do something to help them do well at school?
Educational aspirations and values	If you close your eyes and daydream, how far would you like your son or daughter to go in their studies?	Some parents would prefer their children to stay at home for a long time while others would prefer them to leave home and become independent earlier on. Which would you prefer?

**Table 2:** Response types for the different classes regarding ideas about childhood (Type/Class > 50.0).

V-Test	*p*	Class\ Type	Type\ Class	Global	Variable	Type	N
Modern							
6.78	0.000	85.19	83.64	52.94	What procedures were used to find information?	Active sources	54
6.74	0.000	77.61	94.55	65.69	Information about child-rearing	Yes	67
5.87	0.000	80.36	81.82	54.90	How can intelligence be fostered?	Active procedures	56
4.04	0.000	65.38	92.73	76.47	Can intelligence be fostered?	Yes	78
3.99	0.000	77.27	61.82	43.14	Causes of different temperament	Nature-nurture	44
3.72	0.000	70.00	76.36	58.82	Why do young children play?	To learn	60
3.45	0.000	75.61	56.36	40.20	Education level	High	41
3.04	0.001	62.82	89.09	76.47	Can something be done to make them less shy?	Yes	78
2.83	0.002	69.39	61.82	48.04	The fact that your son or daughter is very masculine/feminine	Least important	49
2.68	0.004	63.77	80.00	67.65	Age at which they should be told off	Under 18 months	69
2.47	0.007	62.86	80.00	68.63	How far will your son or daughter realistically get in his or her studies?	University	70
2.12	0.017	64.29	65.45	54.90	That they be independent	Fairly/the most important	56
1.93	0.027	63.64	63.64	53.92	Sex	Female	55
1.72	0.043	62.50	63.64	54.90	Role of father and mother	The same	56
Traditional–Moderate							
4.25	0.000	55.88	67.86	33.33	When do they start to speak properly?	Between 3 and 4 years	34
3.64	0.000	51.43	64.29	34.31	Information about child-rearing	No	35
3.63	0.000	43.40	82.14	51.96	Causes of different temperament	Nature	53
2.85	0.002	47.06	57.14	33.33	What procedures were used to find information?	No information sought	34
2.83	0.002	41.67	71.43	47.06	Causes of intelligence	Nature	48
2.80	0.003	40.38	75.00	50.98	Sought information about child-rearing	No	52
2.50	0.006	40.43	67.86	46.08	Sex	Male	47
1.95	0.025	34.92	78.57	61.76	Origin of ideas	Experience	63
1.66	0.049	37.21	57.14	42.16	That they show initiative	Fairly important	43
Traditional							
5.14	0.000	65.00	68.42	19.61	What can be done with a shy child?	Don’t know/No answer	20
4.94	0.000	36.54	100.00	50.98	Sought information about child-rearing	No	52
4.93	0.000	73.33	57.9	14.71	Can something be done to make them less shy?	No	15
4.52	0.000	64.71	57.89	16.67	How can intelligence be fostered?	It can’t	17
4.31	0.000	44.12	78.95	33.33	What procedures were used to find information?	No information sought	34
3.66	0.000	40.00	73.68	34.31	Information about child-rearing	No	35
3.42	0.000	42.86	63.16	27.45	Degree of importance: your child should get good grades	The most important	28
3.40	0.000	45.83	57.89	23.53	How far will your son or daughter realistically get in his or her studies?	Lower than university	24
2.55	0.005	62.50	26.32	7.84	Causes of gender differences	Don’t know	8
2.51	0.006	33.33	63.16	35.29	Habitat	Rural	36
2.51	0.006	31.71	68.42	40.20	Why do young children play?	Entertainment/fun	41
2.13	0.017	33.33	52.63	29.41	How confident is your partner?	Very confident	30
2.11	0.017	28.89	68.42	44.12	Role of father and mother	Different	45
1.87	0.031	26.42	73.68	51.96	Degree of importance: that your child be obedient	Fairly/the most important	53
1.82	0.035	27.08	68.42	47.06	Causes of intelligence	Nature	48

**Table 3:** Trajectories of ideas about childhood throughout the longitudinal follow-up.

V-Test	*p*	Class\ Type	Type\ Class	Global	Variable	Type	N
Stable Modern Pattern							
7.78	0.000	94.55	83.87	53.92	Time 4	Modern	55
6.29	0.000	90.38	75.81	50.98	Time 1	Modern	52
6.14	0.000	77.92	96.77	75.49	Time 2	Modern	77
5.64	0.000	83.61	82.26	59.80	Time 3	Modern	61
Stable Traditional–Moderate Pattern							
6.51	0.000	67.86	86.36	27.45	Time 4	Traditional–Moderate	28
5.70	0.000	52.63	90.91	37.25	Time 1	Traditional–Moderate	38
3.78	0.000	52.00	59.09	24.51	Time 2	Traditional–Moderate	35
Changing Traditional/Traditional–Moderate/Traditional–Moderate/Traditional Pattern							
6.66	0.000	100.00	66.67	11.76	Time 1	Traditional	12
6.12	0.000	73.68	77.78	18.63	Time 4	Traditional	19
5.06	0.000	41.46	94.44	40.20	Time 3	Traditional–Moderate	41
2.91	0.000	40.00	55.56	24.51	Time 2	Traditional–Moderate	25

**Table 4:** Response types for the different classes regarding ideas about adolescence (Type/Class > 50.00).

V-Test	*p*	Class\ Type	Type\ Class	Global	Variable	Type	N
Modern							
6.99	0.000	75.86	84.62	28.43	Going out with friends	Totally mature	29
5.98	0.000	100.00	50.00	12.75	Taking decisions about religion	Totally mature	13
5.41	0.000	76.19	61.54	20.59	Choosing their friends	Totally mature	21
5.32	0.000	78.95	57.69	18.63	Having their own ideas about moral issues	Totally mature	19
4.04	0.000	51.43	69.23	34.31	Choosing their own clothes	Totally mature	35
3.30	0.000	45.95	65.38	36.27	Making them do domestic chores	Very important	37
3.16	0.001	36.51	88.46	61.76	Kissing them and showing explicit affection	Very important	63
2.76	0.003	38.78	73.08	48.04	Going out and doing things together	Very important	49
2.70	0.003	45.16	53.85	30.39	Assessing the influence of friends	Positive	31
2.41	0.008	36.54	73.08	50.98	Helping them with schoolwork	Very important	52
Traditional–Moderate							
4.91	0.000	88.24	54.55	33.33	Having their own ideas about moral issues	Moderately mature	34
4.14	0.000	82.86	52.73	34.31	Making them do domestic chores	Important	35
3.70	0.000	74.47	63.64	46.08	Going out with friends	Mature	47
3.21	0.001	73.81	56.36	41.18	When prefer child to become independent	Don’t know	42
2.81	0.002	74.29	47.27	34.31	Going out and doing things together	Fairly important	35
2.71	0.003	57.89	100.00	93.14	Indecisive about studies. Who should intervene?	Father and mother	95
2.47	0.007	62.86	80.00	68.63	Is adolescence a very difficult age?	Yes	70
Traditional							
3.50	0.000	52.38	52.38	20.59	Having sexual intercourse	Totally immature	21
2.91	0.002	44.00	52.38	24.51	When prefer child to become independent	Later	25
2.89	0.002	34.04	76.19	46.08	Sex	Male	47
2.73	0.003	35.90	66.67	38.24	Having a girlfriend/boyfriend	Immature	39
2.59	0.005	33.33	71.43	44.12	Ideal age for children to leave home	25 years and over	45

**Table 5:** Association between parental ideas about childhood and ideas about adolescence in percentage terms.

			Childhood	
Adolescence		Modern	Traditional–Moderate	Traditional
	Modern	14.8 **	5.1	6.1
	Traditional–Moderate	28.4	12.7	12.7
	Traditional	6.9 **	3.9	9.8 **
		Evolution of Ideas about Childhood		
Adolescence		Modern	Traditional–Moderate	Traditional
	Modern	20.6 **	3.9	1.0 **
	Traditional–Moderate	32.4	11.8	9.8
	Traditional	7.8 **	5.9	6.9 **

**p<0.01; Adjusted standardized residuals > ±2.4 are shared.

## Data Availability

Not applicable.
